# Population-Based Study on the Effect of a Forest Environment on Salivary Cortisol Concentration

**DOI:** 10.3390/ijerph14080931

**Published:** 2017-08-18

**Authors:** Hiromitsu Kobayashi, Chorong Song, Harumi Ikei, Bum-Jin Park, Juyoung Lee, Takahide Kagawa, Yoshifumi Miyazaki

**Affiliations:** 1 Department of Nursing, Ishikawa Prefectural Nursing University, 1-1 Gakuendai, Kahoku, Ishikawa 929-1210, Japan; kobayasi@ishikawa-nu.ac.jp; 2 Center for Environment, Health, and Field Sciences, Chiba University, 6-2-1 Kashiwa-no-ha, Kashiwa, Chiba 277-0882, Japan; crsong1028@chiba-u.jp (C.S.); ikei0224@ffpri.affrc.go.jp (H.I.); 3 Forestry and Forest Products Research Institute, 1 Matsunosato, Tsukuba, Ibaraki 305-8687, Japan; kagawa@ffpri.affrc.go.jp; 4 Department of Environment and Forest Resources, Chungnam National University, 99 Daehak-ro, Yuseong-gu, Daejeon 34134, Korea; bjpark@cnu.ac.kr; 5 Department of Landscape Architecture, Hankyong National University, 327 Jungang-ro, Anseong-si, Gyeonggi-do 17579, Korea; lohawi@gmail.com

**Keywords:** forest environment, salivary cortisol, distribution, population approach

## Abstract

The purpose of this study was to evaluate the effect of a forest environment on salivary cortisol concentration, particularly on the characteristics of its distribution. The participants were 348 young male subjects. The experimental sites were 34 forests and 34 urban areas across Japan. The subjects viewed the landscape (forest or urban environment) for a period of 15 min while sitting in a chair. Saliva was sampled from the participants at the end of this 15-min period and then analyzed for cortisol concentration. Differences in the skewness and kurtosis of the distributions between the two environments were tested by performing a permutation test. The cortisol concentrations exhibited larger skewness (0.76) and kurtosis (3.23) in a forest environment than in an urban environment (skewness = 0.49; kurtosis = 2.47), and these differences were statistically significant. The cortisol distribution exhibited a more peaked and longer right-tailed curve in a forest environment than in an urban environment.

## 1. Introduction

There has been growing interest in the beneficial effects of the natural environment on human health. Positive responses to the natural environment have been demonstrated in various studies. Bowler [1] reviewed studies on the beneficial effects of natural environments and concluded that walking or running in natural environments reduced self-reported negative emotions such as anger, fatigue, and sadness. It has also been shown that patients with exhaustion disorder perceived greater restorative and mood-enhancing effects as well as improved capacity for attention in forest environments compared to urban environments [2]. The effects of natural environments on affect, cognition, and physiology have been well established in laboratory and field experiments [3]. Rooftop green spaces may also have beneficial effects, similar to those of outdoor natural environments, for elderly patients who require long-term care [4]. Furthermore, a large study observed lower mortality (especially due to cardiovascular diseases) in populations with greater exposure to green space, and this tendency was independent of income level [5].

In addition to psychological conditions, the stress-reducing effects of a natural environment have been evaluated by using various physiological functions. Heart rate variability (HRV) is a representative stress indicator that has been used to evaluate the effects of exposure to natural environments. In our previous study of healthy male students, the high-frequency component of HRV (HF) was significantly larger and the low-to-high-frequency component ratio (LF/HF) was smaller in a forest environment than in an urban environment. The HF and LF/HF are considered to be indicators of parasympathetic activity and sympatho-vagal balance, so the results suggested that forest environments induce autonomic relaxation [6]. Richardson et al. [7] performed a meta-analysis that included 13 relevant studies and estimated the effect size of forest environments on HF to be about 0.71 (Hedge’s g). This could be interpreted as a medium to large effect.

Along with HRV, salivary cortisol measurement has been used as a neuroendocrine stress indicator. Cortisol is a steroid hormone produced by the zona fasciculata of the adrenal cortex to regulate carbohydrate, fat, and protein metabolism. The cortisol level is considered to be an indicator of the activity of the hypothalamic-pituitary-adrenal (HPA) axis, which is responsible for corticotropin-releasing hormone (CRH) and adrenocorticotropin (ACTH) secretions. The HPA system is among the major stress response systems in humans. ACTH-mediated cortisol secretion induces increases in blood pressure and glucose levels as well as suppression of the immune system. These physiological reactions are called the “fight or flight” response. Thus, cortisol measurements have been used as a biological indicator of stress. In recent years, several attempts have been made to assess the physiological effects of natural environments by measuring cortisol.

The authors have previously demonstrated lower salivary or serum cortisol concentrations in response to forest environment exposure [8,9,10,11]. Similarly, Mao et al. [12] reported a decrease in serum cortisol levels in forest environments. Thompson et al. [13] reported that people who had more access to green space exhibited a steeper decline in salivary cortisol concentration from morning to afternoon; however, their results did not reveal any significant difference in the mean cortisol concentrations. A similar steeper decline in cortisol during exposure to a forest environment was reported in hypertensive elderly patients [14].

Conversely, other studies have reported insignificant changes in mean salivary cortisol levels during viewing or walking in a forest. A study conducted in Finland failed to find a significant difference in salivary cortisol levels between exposure to urban and green areas [15], and other studies also found statistically insignificant relationships between natural environments and salivary cortisol [16,17]. These results indicate that no consensus has yet been reached on the effect of forest environments on salivary or serum cortisol concentration.

There are two types of strategies for health promotion: a high-risk (individual) approach and a population approach. The high-risk approach targets individuals with a certain disease or impairment, and the population approach targets a whole population. Although its effect on each individual is relatively small, the population approach can achieve greater health improvement as a whole by shifting the risk distribution curve of the population [18]. Nature therapy, including “forest bathing” is one population approach for health promotion; thus, its effects should be evaluated by a population-based analysis. However, most of the previous studies on natural therapy have merely focused on the change in the mean values of health-related variables. Therefore, the present study investigated the effect of a forest environment on salivary cortisol concentration in 348 male participants, with an emphasis on the characteristics and shape of the distribution of these values.

## 2. Materials and Methods

### 2.1. Participants

A total of 408 Japanese male students participated in this study, and 372 of them completed the experiment. None of the participants reported a history of physical or psychiatric disorders. Alcohol and tobacco consumption was prohibited, and caffeine consumption was controlled during the study period. On the day before the experiments, the participants were fully informed of the study aims and procedures and their informed consent was obtained. They were paid 8800 yen (about US$80) per day for their participation in the study. The study was conducted under the regulations of the Institutional Ethical Committee of the Forestry and Forest Products Research Institute, Japan (project identification code number: 16-558), or the Center for Environment, Health, and Field Sciences, Chiba University, Chiba, Japan.

### 2.2. Experimental Procedures and Salivary Cortisol Measurement

The study was designed as a random crossover trial. The experimental sites were 34 forest and 34 urban areas across Japan. The experiments were conducted on weekdays from early July to early September. Twelve males participated in each experiment. The chosen urban sites were in city centers or near a Japanese Railway station. The experiment was performed at each experimental site over two consecutive days.

The participants were randomly divided into two groups. One group was exposed to a forest site prior to an urban site, and the reverse order was applied to the other group. All participants remained in a waiting room before moving to the field site. The participants rested on chairs and viewed the landscape for 15 min, during which time they were instructed to stay awake. After this, their saliva was sampled individually. After the experiment on the first day, the participants all stayed for the night at the same hotel with identical single rooms.

On the second day, the participants changed to the other experimental environment. The experimental protocol on the second day was the same as that on the first day. Salivary samples were collected before lunch. The time of saliva collection varied from 9:30 to 12:00 am. Although the time of saliva collection varied among the participants, in both environments each participant was measured at approximately the same time on each experimental day.

Saliva was collected by using Salivette^®^ (No. 51.1534; Sarstedt, Nümbrecht, Germany). The participants held two pieces of absorbent cotton in their mouth for 2 min, and the saliva in the wads was later extracted by centrifugation. The samples were immediately frozen and transported to the laboratories of SRL, Inc. (Tokyo, Japan). Each sample consisted of a 0.25-mL aliquot of saliva, and its cortisol concentration was analyzed by radioimmunoassay.

This study was part of a large-scale research project on the effects of the forest environment on psychological and physiological functions. In addition to salivary cortisol, the broader project investigated heart rate variability, blood pressure, and the Profile of Mood States (POMS). In the present study, we only consider the results that focused on the change in the distribution characteristics (but not the average values) of the concentrations of salivary cortisol. The results and detailed measurement procedures for the other indicators have been reported elsewhere [19,20].

### 2.3. Outlier Processing

Outlier processing was performed on the results because higher-moment statistics (skewness and kurtosis) are particularly sensitive to outliers [21]. The outlier processing was based on a box-whisker plot [22]. Upper and lower cutoffs (UpperCO and LowerCO) were defined as follows:

UpperCO = Q3 + 1.5 (IQR),(1)

LowerCO = Q1 − 1.5 (IQR),(2)

where

Q1: quartile 1 (25th percentile)
Q3: quartile 3 (75th percentile)
IQR: interquartile range (Q3–Q1)

The outlier processing was performed on the results obtained in each environment (urban and forest); lower outliers were not observed because LowerCOs exhibited negative values in both environments. The UpperCOs were 15.31 and 18.90 nmol/L for the urban and forest environments, respectively. The participants associated with outliers in either or both environments were eliminated. As a result, 24 participants were eliminated and the data of the remaining 348 participants were used for further analysis. The demographic parameters of the 348 participants are shown in Table 1.

### 2.4. Statistical Analysis

Salivary cortisol concentrations of the 348 participants were plotted as histograms by dividing the range from 0 to 18 nmol/L into 22 segments. Changes in salivary cortisol concentrations between urban and forest environments (forest-urban) were also plotted as a histogram by dividing the range from −12 to +12 nmol/L into 22 segments. The mean, median, standard deviation (SD), coefficient of variation (CV), IQR, skewness, and kurtosis of the distribution were calculated. Skewness is a measure of the symmetry of distribution. Negative or positive skewness is indicated when the left or right tail of the research data in a histogram is longer than the other tail. The skewness of a normal distribution is zero. In addition, kurtosis is a measure of whether the distribution curve is peaked (positive) or flat (negative) relative to the normal distribution. The kurtosis of normally distributed data is defined as 3. Differences in these statistics between urban and forest environments were tested by performing a permutation test, which is a statistical test with a non-parametric basis. Resampling was performed 5000 times. The *p*-value was calculated according to the suggestion by Phipson and Smyth [23]. *p*-values of <0.05 were considered as indicating statistical significance. The uncertainty of a *p*-value near 0.05 was estimated to be 0.3%.

## 3. Results

**Figure 1:**
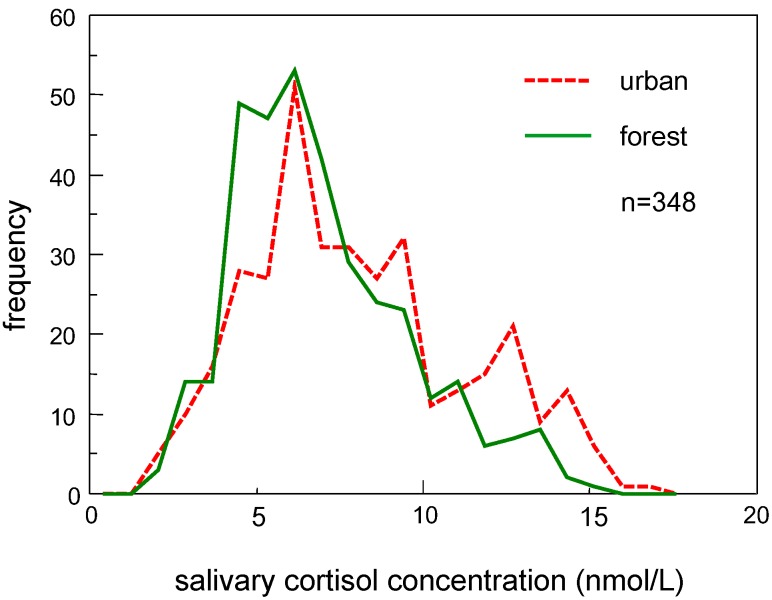
Histogram showing the salivary cortisol concentrations in urban and forest environments. The frequency of subjects with a higher cortisol concentration was lower in a forest environment compared to that in an urban environment.

Frequency distributions of salivary cortisol concentrations were plotted as a histogram, as shown in Figure 1. Mode (the bin of cortisol concentrations that included the largest number of participants) was in the range from 5.73 to 6.55 nmol/L for both environments; however, the number of individuals with a higher cortisol concentration (>10 nmol/L) was larger in an urban environment (*n* = 84) than in a forest environment (*n* = 44). The statistics for both distributions and the results of the permutation tests are summarized in Table 2. Cortisol concentrations were significantly lower in a forest environment (mean: 6.88 nmol/L, median: 6.35 nmol/L) than in an urban environment (mean: 7.98 nmol/L, median: 7.45 nmol/L), and the permutation test revealed that these differences were statistically significant (*p* < 0.001). In addition to the mean and median, a significantly smaller SD (*p* < 0.001) was observed for the forest environment data. A smaller IQR was also observed in a forest environment, although the difference was not statistically significant (*p* = 0.123).

As for the higher moment statistics, larger skewness and kurtosis (0.76 and 3.23, respectively) were observed in a forest environment than in an urban environment (0.49 and 2.47, respectively), and the differences were statistically significant (*p* = 0.037 and *p* = 0.017, respectively). The larger skewness and kurtosis indicated that the distribution was peaked and had a longer right-tailed curve in a forest environment.

**Figure 2:**
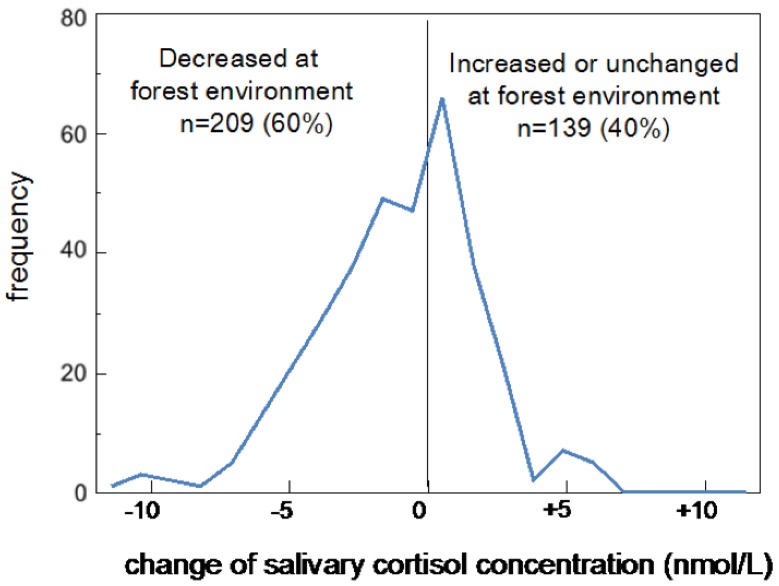
Histogram showing the differences in cortisol concentrations between urban and forest environments. Approximately 60% (*n* = 209) of the participants exhibited decreased cortisol concentrations in a forest environment, and the remaining 40% (*n* = 139) exhibited increased (*n* = 118) or unchanged (*n* = 21) cortisol concentrations.

The distribution of the differences in cortisol concentrations between urban and forest environments was also plotted as a histogram (Figure 2). Positive and negative values represent increases and decreases in cortisol in a forest environment, respectively. The differences ranged from −6.35 to +11.59 nmol/L, and the mode was in the range from 0 to +1.09 nmol/L (*n* = 66), which included 21 participants who exhibited the same cortisol level in both environments. Approximately 60% (*n* = 209) of the participants exhibited decreases in a forest environment, and the remaining 40% (*n* = 139) exhibited increased or unchanged cortisol concentrations.

## 4. Discussion

As mentioned previously, some researchers have reported insignificant changes in or increased concentrations of cortisol upon exposure to forest environments. On the other hand, the present study demonstrated significantly lower salivary cortisol concentrations upon exposure to forest environments than to urban environments. The present results on the changes in mean concentration were almost identical to our previous results [20]. Except for those in epidemiological investigations, such as a study by Mitchell and Popham [5], the dataset analyzed in the present study may be the largest yet in experimental studies on the effect of forest environments on cortisol concentrations.

The authors previously investigated the physiological effects of forest environments on the frequency components of HRV in 625 male subjects [6]. Approximately 79% of the subjects had increased HF upon exposure to forest environments, and the remaining 21% had opposite responses. Additionally, 64% of the previous subjects had decreases in LF/HF and 34% had increases upon exposure to forest environments. On the other hand, the ratio of positive and negative responders to the forest was approximately 60:40 in the present study on salivary cortisol. A comparison of the results of HRV and salivary cortisol showed that HRV (especially HF) might be more responsive to natural environments than salivary cortisol.

The present study demonstrated significantly larger skewness and kurtosis of the cortisol distribution in forest environments. Exposure to forest environments modified the distribution curve of salivary cortisol concentrations in addition to causing a significant change in the mean salivary cortisol concentration. On the other hand, in our previous study, the parasympathetic indicator (HF) of HRV increased in forest environments, whereas the skewness and kurtosis of the distribution were not clearly changed [6]. In short, the forest environments shifted the distribution curve of HF to a higher value while maintaining the shape of the curve. In this respect, the responses of cortisol and HRV to forest environments were different, although significant changes in the mean values of both biological indicators were observed. Therefore, analysis of the characteristics of the data distribution revealed another aspect of the physiological effects of nature therapy.

Cortisol is known to exhibit a large diurnal variation. Salivary cortisol concentrations increase after awakening, peaking at approximately 30 min after awakening in the morning and gradually decreasing throughout the day. This phenomenon is known as the cortisol awakening response [24]. The peak concentration of salivary cortisol has been reported to be approximately 16–21 nmol/L [25,26,27,28,29]. In the present study, saliva samples were collected from 9:30–12:00 am., and the mean cortisol concentrations were approximately 7–8 nmol/L. Lower cortisol concentrations are expected when measured in the afternoon. If the baseline (urban environments) value is too low, it might be difficult to accurately assess decreases caused by exposure to forest environments. In fact, saliva that was sampled in the afternoon in previous studies demonstrated non-significant effects of urban and forest environments on cortisol concentrations [15,16]. Brandtstädter et al. [30] studied the effects of socioeconomic status, such as income, education, employment, and occupation, on salivary cortisol level, and reported that the effect was clear in the morning but not in the afternoon and evening. These previous results support the above hypothesis. The difference in time of day for saliva collection might be a reason for inconsistent results on the effect of natural environments on cortisol concentration.

Various stresses in modern life, such as noise from traffic [31], crowded spaces [32], shift work [33], and lower socioeconomic status [30], cause an increase in cortisol. Chronic exposure to elevated cortisol can cause various pathogenic processes, including cognitive decline, immunosuppression, and insulin resistance [34]. Therefore, lowering cortisol levels may help prevent various diseases. “Forest bathing” is considered to be an effective intervention as a population approach to health promotion.

## 5. Conclusions

A novel finding of this study was that salivary cortisol concentrations demonstrated greater skewness and kurtosis in forest environments than in urban environments. Exposure to forest environments modified the distribution curve of salivary cortisol concentrations in addition to causing a significant change in the mean salivary cortisol concentration. Although previous studies have shown decreases in mean cortisol concentration in forest environments, no previous study has focused on the distribution characteristics of salivary cortisol concentration. This population-based analysis provides a new perspective on the physiological effect of natural environments.

## Figures and Tables

**Table 1:** Demographics of the participants (*n* = 348).

Variable	Age (Year)	Height (m)	Body Mass (kg)
max	28	1.87	104
min	20	1.58	42
mean	21.7	1.72	64.2
SD	1.6	0.05	9.7

SD: standard deviation.

**Table 2:** Distribution characteristics of salivary cortisol measurements in urban and forest environments.

Variable	Urban Environment	Forest Environment	Difference
	*n* = 348	*n* = 348	
mean (nmol/L)	7.98	6.88	*p* < 0.001
median (nmol/L)	7.45	6.35	*p* < 0.001
SD (nmol/L)	3.33	2.75	*p* < 0.001
CV (%)	40.8	38.5	*p* = 0.206
Q1	5.79	4.97	*p* = 0.001
Q3	9.93	8.55	*p* < 0.001
IQR (nmol/L)	4.14	3.59	*p* = 0.123
skewness	0.49	0.76	*p* = 0.037
kurtosis	2.47	3.23	*p* = 0.017

SD: standard deviation; CV: coefficient of variation; Q1: quartile 1 (25th percentile); Q3: quartile 3 (75th percentile); IQR: interquartile range; Skewness: a measure of symmetry of distribution; Kurtosis: a measure of whether the distribution curve is peaked (positive) or flat (negative) relative to the normal distribution. Differences between urban and forest environments were tested by a permutation test.
